# DV3-IBi_YOLOv5s: A Lightweight Backbone Network and Multiscale Neck Network Vehicle Detection Algorithm

**DOI:** 10.3390/s24123791

**Published:** 2024-06-11

**Authors:** Liu Wang, Lijuan Shi, Jian Zhao, Chen Yang, Haixia Li, Yaodong Jia, Haiyan Wang

**Affiliations:** 1 Key Laboratory of Intelligent Rehabilitation and Barrier-Free for the Disabled, Ministry of Education, Changchun University, Changchun 130022, China; wangl95@ccu.edu.cn (L.W.); zhaojian@ccu.edu.cn (J.Z.); 210701225@mails.ccu.edu.cn (C.Y.); lihx0621@163.com (H.L.); jiayaodong2024@126.com (Y.J.); wanghy80@ccu.edu.cn (H.W.); 2 Jilin Provincial Key Laboratory of Human Health Status Identification & Function Enhancement, Changchun 130022, China; 3 College of Computer Science and Technology, Changchun University, Changchun 130022, China; 4 College of Electronic and Information Engineering, Changchun University, Changchun 130022, China

**Keywords:** vehicle detection, lightweight, multiscale, YOLOv5s

## Abstract

Vehicle detection is a research direction in the field of target detection and is widely used in intelligent transportation, automatic driving, urban planning, and other fields. To balance the high-speed advantage of lightweight networks and the high-precision advantage of multiscale networks, a vehicle detection algorithm based on a lightweight backbone network and a multiscale neck network is proposed. The mobile NetV3 lightweight network based on deep separable convolution is used as the backbone network to improve the speed of vehicle detection. The icbam attention mechanism module is used to strengthen the processing of the vehicle feature information detected by the backbone network to enrich the input information of the neck network. The bifpn and icbam attention mechanism modules are integrated into the neck network to improve the detection accuracy of vehicles of different sizes and categories. A vehicle detection experiment on the Ua-Detrac dataset verifies that the proposed algorithm can effectively balance vehicle detection accuracy and speed. The detection accuracy is 71.19%, the number of parameters is 3.8 MB, and the detection speed is 120.02 fps, which meets the actual requirements of the parameter quantity, detection speed, and accuracy of the vehicle detection algorithm embedded in the mobile device.

## 1. Introduction

The advent of the information age has promoted the development of intelligent vehicles, intelligent travel, traffic safety, and accident analysis. Traditional traffic monitoring equipment can record a large amount of video vehicle information, mainly collecting vehicle flow and license plate information, but this method has difficulty meeting the needs of traffic intelligence. To monitor and protect traffic roads more effectively and maintain social stability, vehicle detection technology is needed to detect the location and attributes of vehicles on roads quickly and accurately.

Traditional vehicle recognition technology uses classical classifiers and manually designed feature methods, while vehicle detection technology uses image background modeling and positioning, relying on the manual selection of prior feature vehicles and classification [1,2,3,4,5,6]. This method has several limitations, such as high data requirements, slow detection speed, and poor generalization ability, which cannot meet the needs of traffic intelligence. In contrast, the rise of deep learning provides strong support for image recognition technology and target detection, especially in the fields of intelligent driving and machine learning. That is, the target detection algorithm based on deep learning is more suitable for vehicle detection scenarios and has become the mainstream algorithm in this field [7,8,9,10]. The vehicle detection algorithm mainly focuses on the two key subtasks of vehicle location and classification and focuses on improving the accuracy of detection and the diversity of classification. Vehicle detection algorithms based on deep learning can realize real-time detection and tracking of vehicles to improve the efficiency of traffic management and safety monitoring. Research on vehicle detection algorithms based on deep learning is very important for promoting the development of intelligent transportation systems and improving traffic safety. The YOLO algorithm can recognize the exact position and category of objects in a graph only by browsing once. In recent years, YOLO series algorithms have been gradually improved and are widely used in target detection research [11,12,13,14,15].

Using the YOLO algorithm to realize vehicle detection has become the main research direction. In research on improving the accuracy of vehicle detection, Zhang [16] improved the YOLOv2 framework model, optimized the model parameters, expanded the grid size, increased the number and size of anchor points in the model, and realized high-precision and real-time automatic vehicle detection and vehicle category recognition. Compared with that of the fast RCNN, the accuracy rate increased to 91.80%, and the recall rate increased to 63.86%. Hamzenejadi [17] improved the YOLOv5s model, added an attention mechanism and adaptive boundary box regression loss function, balanced the depth and width of the model, and incorporated convolution into the neck unit to further improve the balance between accuracy and speed. Mittal [18] proposed the hybrid model of faster R-CNN and YOLO. Compared with the basic estimator on the collected dataset, the hybrid model is better than YOLO and faster R-CNN in estimating traffic density.

In vehicle detection research on lightweight networks, Shao [19] proposed a lightweight UAV image target detection method based on yolov8; Taheri [20] proposed an improved tiny yolov3 method, which takes a tiny yolov3 network as the basic reference model and discards unnecessary layers through pruning and simplification on the bit vehicle dataset, effectively improving the accuracy and speed of the detection model. Dong [21] improved the lightweight YOLOv5 vehicle detection method, introduced c3ghost and ghost modules to reduce floating-point operations (FLOPs) in the process of feature channel fusion, enhanced the performance of feature expression, and introduced a convolution block attention module (CBAM) to suppress irrelevant information and improve the detection accuracy. According to the relevant datasets, the accuracy of the proposed model increased by 3.2%, the number of flops decreased by 15.24%, and the number of model parameters decreased by 19.37%. Ge [22,23,24] improved the Yoyov3 model by using lightweight networks such as the darknet-19 and ResNet-18 networks to realize feature extraction and training and testing the Kitti datasets, and compared with the traditional Yoyov3 dataset, the average accuracy increased by 14.09%, reaching 93.66%.

Therefore, in the vehicle detection task, the optimized loss function is used to enhance the positioning accuracy, and the flipped splicing method is used to improve the extraction of small target features from the network and improve the detection performance. By adjusting the network depth or using separable convolution, although some detection accuracy will be lost, this approach speeds up the detection speed and is more suitable for embedded devices and other resource-limited environments. In a scene with dense vehicles, the aggregation module can also be used to enhance the feature extraction ability. Improving the detection accuracy of the YOLO algorithm for vehicle detection and using a lightweight network to improve the speed of the detection model are the main research directions at this stage. Therefore, this study proposes a vehicle detection algorithm consisting of a lightweight backbone network and a multiscale neck network, which aims to improve the speed of vehicle detection by optimizing the backbone network and adapting to the memory capacity of mobile devices. Additionally, by optimizing the neck network, the vehicle detection accuracy at night and in complex scenes can be improved to meet the dual real-time and accuracy requirements.

## 2. Lightweight Vehicle Detection Algorithm Based on Mobilenetv3

In the training of deep learning models, processing a large amount of data is a common requirement. It is a challenging task to accelerate the calculation without reducing the quality of data processing. To solve this problem, the MobileNet series of lightweight models was developed. Mobilenetv3 can achieve efficient computing in resource-constrained environments such as mobile devices. However, the algorithm has insufficient ability to understand the global information of the input image, which will lead to the problem of decreasing the accuracy of specific vehicle detection [25].

The design of MobileNetv3 focuses on lightweight and efficient performance. When applied to vehicle detection, deep separable convolution and pointwise convolution in MobileNetv3 may lead to the loss of spatial information [26,27]. Large-scale vehicle features are distributed throughout the whole image space, but the design of MobileNetv3 cannot capture this spatial information well, which affects the recognition performance of complex vehicles. To solve this problem, this study combines the advantages of hole convolution and deep separable convolution and builds a MobileNetv3 model based on deep hole separable convolution (DDSC_V3), which expands the receptive field area, avoids the introduction of redundant parameters, and improves the efficiency of network detection.

### 2.1. Backbone Network Based on DDSC_V3

**Figure 1:**
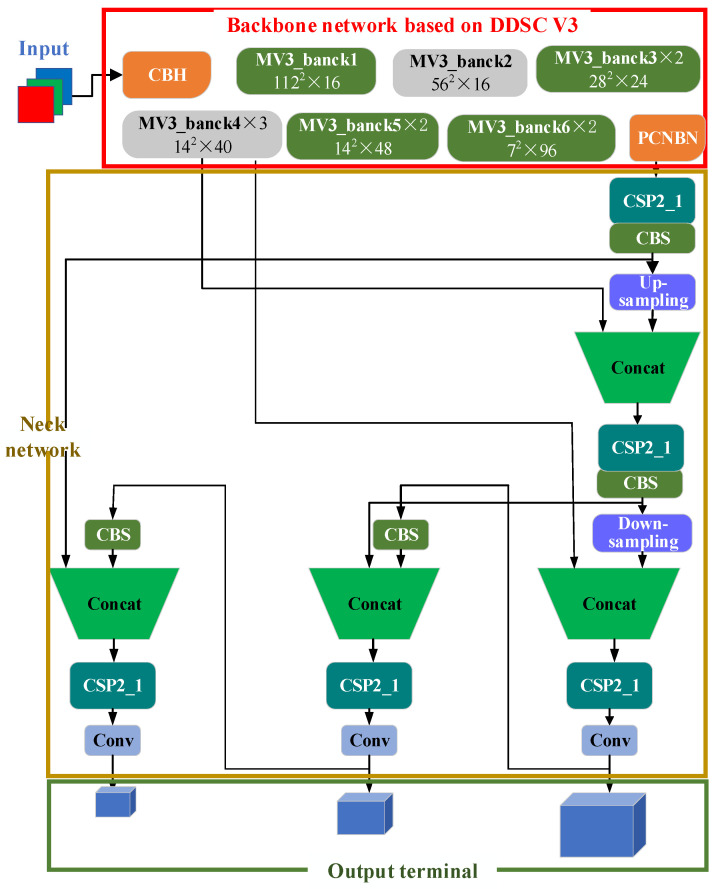
Model diagram of the DDSC_v3_YOLOv5s algorithm.

The backbone network based on DDSC_V3 is built on the inverted residual block of the MobileNetv3 network and consists of eight parts, as shown in Figure 1. Convolution, batch normalization, and hard swish (CBH) in the figure include convolution, batch normalization (BN), and hard swish; the input of the mv3_bank1 module is 112 × 112 × 16, and the convolution kernel is 3 × 3; Mv3_bank2 is the input 56 × 56 × 24, and the convolution kernel is 3 × 3; the input of mv3_bank3 is 28 × 28 × 24, and the convolution kernels are 3 × 3 and 5 × 5; the input of mv3_bank4 is 14 × 14 × 40, and the convolution kernel is 5 × 5; the input of mv3_bank5 is 14 × 14 × 48, and the convolution kernel is 5 × 5; the input of MV3_bank6 is 7 × 7 × 96, and the convolution kernel is 5 × 5; and PNC contains convolution, pooling, and nonlinear activation functions. The ddsc_v3 backbone network is responsible for extracting the high-level features of the input image for subsequent vehicle detection tasks [28].

### 2.2. Cervical Network

In Figure 1, the neck network uses a series of convolution layers and pooling layers to gradually reduce the size of the feature map and increase the number of feature channels to improve the perception and presentation ability of the network. It is responsible for extracting features and reducing the spatial dimension of the feature graph. In Figure 1, the CBS is conv+bn+silu. First, the convolution layer is applied, and then the convolution result is a BN. Finally, the nonlinear transformation is carried out through the sigmoid activation function, and the convergence process of the model is accelerated through batch normalization to help the network better learn the network characteristics. CSP2_X is composed of CBS and residual components. The input is divided into two branches. One branch directly performs convolution calculations, and the other branch performs convolution calculations of the CBS residual structure (bank × n). Then, the two branches are superimposed and fused: BN normal distribution, reactivation, and CBS.

### 2.3. Output Terminal

In Figure 1, the output layer mainly includes the convolution layer of the prediction target frame and category probability. The final number of output channels is the probability and category of the target. The output layer uses a sigmoid activation function to predict the category probability of the coordinate information (x, y, W, H).

To obtain a larger receptive field, the DDSC_V3_YOLOv5s network proposed in this article uses deep hole separable convolution and a MobileNetv3 lightweight network to complete the rapid feature extraction task of the backbone network. In the neck network and output layer, the same strategy as YOLOv5s is adopted to realize vehicle detection.

## 3. Multiscale Vehicle Detection Algorithm Based on an Attention Mechanism and Pyramid Theory

### 3.1. Construction of the Two-Way Cross-Scale Feature Pyramid

The traditional feature pyramid network is usually a bottom-up one-way structure, while bifpn introduces top-down information flow so that the feature information can be spread and adjusted in two directions to better integrate the semantic information at different levels [29,30,31].

The backbone network module extracts feature information tensors of different scales on different convolution layers, expressed as Formula (1):

(1) $${\vec{P}}^{in}=(P_{l1}^{in},P_{l2}^{in},\ldots P_{li}^{in})$$

where $P_{li}^{in}$ is the feature extracted during the convolution operation at layer $l_{i}$.

To aggregate the feature information of different scales, the feature fusion function f is used to aggregate the feature information and output a new tensor feature:

(2) $${\vec{P}}^{out}=f({\vec{P}}^{in})$$

After fusing the different scale features of different convolution outputs in the backbone network, a new multiscale module is obtained.

The feature tensor of the 4–6 fusion layers is taken as an example, that is, ${\vec{P}}^{in}=({{\vec{P}}_{4}}^{in},\ldots ,{{\vec{P}}_{6}}^{in})$, where ${\vec{P}}^{in}$ is the feature layer of the input image 1/2I. When the size of the input image is 640 × 640, ${{\vec{P}}_{6}}^{in}$ is the feature tensor size of the sixth layer, which is 5 × 5 (640/26 = 5), and ${{\vec{P}}_{4}}^{in}$ is the feature tensor size of the fourth layer, which is 80 × 80

(3) $$\begin{array}{l} {{\vec{P}}_{6}}^{out}=Conv({{\vec{P}}_{6}}^{in})\\ {{\vec{P}}_{5}}^{out}=Conv({{\vec{P}}_{5}}^{in}+Rs({{\vec{P}}_{6}}^{out}))\\ {{\vec{P}}_{4}}^{out}=Conv({{\vec{P}}_{4}}^{in}+Rs({{\vec{P}}_{5}}^{out})) \end{array}$$

where $R_{s}$ is the multiscale coefficient. $R_{s}$ is adjusted through upsampling or downsampling to match the corresponding size.

The feature fusion of layer 6 is taken as an example, as shown in Formula (4):

(4) P6td=Convw1⋅P6td+Resize(P7in)w1+w2+∈P6out=Convw′1⋅P6in+w′2⋅P6td+w′3⋅Resize(P5out)w′1+w′2+w′3+∈

where $P_{6}^{td}$ a represents the middle feature of the sixth layer and $P_{6}^{out}$ represents the output characteristics of layer 6. The feature construction method of the other layers is the same as the principle of Formulas (1)–(4).

**Figure 2:**
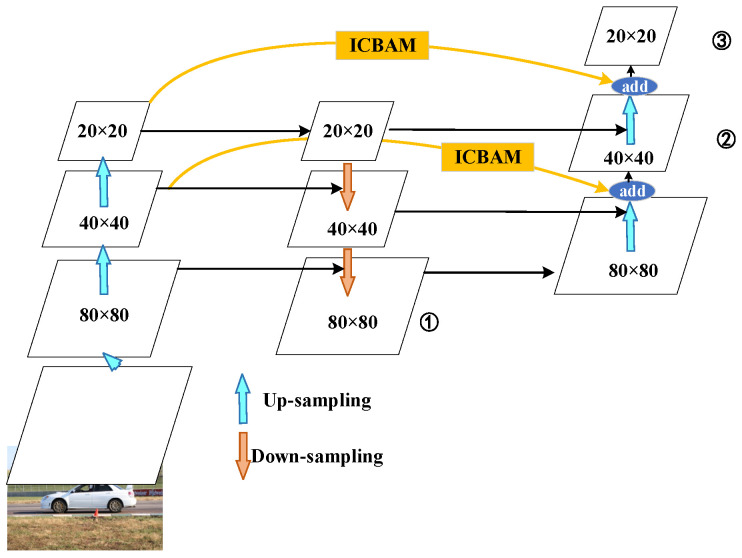
Schematic diagram of ICBAM_BiFPN multiscale feature fusion.

Based on the original YOLOv5s model, taking advantage of the characteristics of the efficient two-way cross-scale connection of bifpn, the simple pan is replaced by bifpn and integrated with the icbam attention mechanism module on the jump path, which is called icbam_bifpn, as shown in Figure 2. The blue arrow in the figure shows the upsampling process; the orange arrow indicates downsampling; and ①, ②, and ③ are the final output characteristic tensor scales of 80 × 80, 40 × 40, and 20 × 20, respectively. The yellow jump path is an improved part of this article.

The yellow area in Figure 2 is the icbam_bifpn algorithm proposed in this section. Compared with those of the original YOLOv5 algorithm, two feature jump fusion paths are added, and an icbam attention mechanism is added to the jump fusion path to obtain different size features while preserving the spatial and channel position information of the detection target to improve the detection accuracy.

### 3.2. YOLOv5s Vehicle Detection Model Based on ICBAM and BiFPN

**Figure 3:**
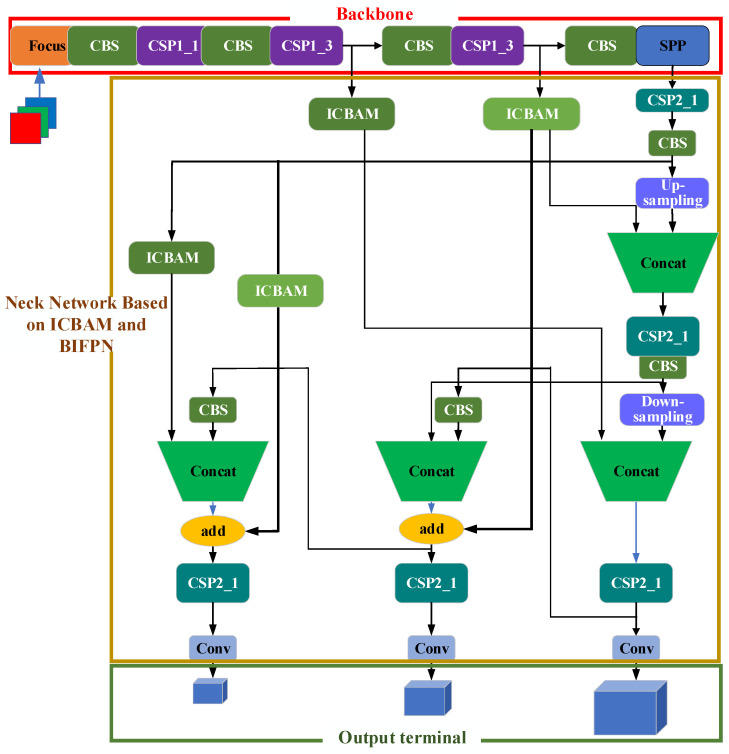
Model diagram of the Ibi_YOLOv5s algorithm.

Combining icbam and icbam_bifpn, a multiscale vehicle detection method using yolov5s based on icbam and bifpn (IBi_YOLOv5s) is proposed, which is mainly divided into three parts: a backbone network, a neck network based on icbam and bifpn (abbreviated as the IBi-based neck network), and an output terminal, as shown in Figure 3.

(1) Backbone network

YOLOv5s uses cspparknet53 as its backbone network, which is composed of 53 convolution layers. The main feature of darknet53 is its moderate depth and width, which can not only effectively extract features from images but also avoid excessive consumption of computing resources due to its complexity. The CSP structure is the key component of cspparknet53. It divides the network into two paths: one path is used to extract features and the other path continues to transfer the original input, and then the two are combined again, which reduces the burden of feature propagation while maintaining the smoothness of the information, making the network more efficient.

(2) Neck network based on IBi

Figure 3 shows the proposed neck network based on IBi. In this paper, four icbam attention mechanism modules and two hopping lines with icbam are added in the process of transmitting characteristic tensors between the backbone network and neck network. The 40 × 40 and 20 × 20 feature tensors obtained through the backbone network directly participate in the feature fusion of the final output feature tensors and use the “add” operation. By adding the icbam attention mechanism module and jump connection between the backbone network and the neck network, the feature information of different scales can be effectively transmitted and integrated, which helps to improve the ability of the network to represent the features of different scales and semantics and increases the accuracy of the model in detecting objects of various sizes and categories.

(3) Output terminal

In Figure 3, the output layer mainly includes a convolution layer for predicting the target frame and category probability. The final number of output channels is the probability and category of the target. The output layer uses a sigmoid activation function to predict the category probability of the coordinate information (x, y, W, H) [32].

The Ibi_YOLOv5s network proposed in this article uses an icbam attention mechanism and a bifpn two-way cross-scale pyramid to add jump connections in the process of feature transmission, reduce the loss of information in the process of feature transmission, and promote the flow and fusion of features to maintain the richness and diversity of feature maps and improve the detection performance of the model. The same strategy used for YOLOv5S is adopted for the backbone network and the output layer.

## 4. Vehicle Detection Algorithm Based on a Lightweight Backbone Network and a Multiscale Neck Network

**Figure 4:**
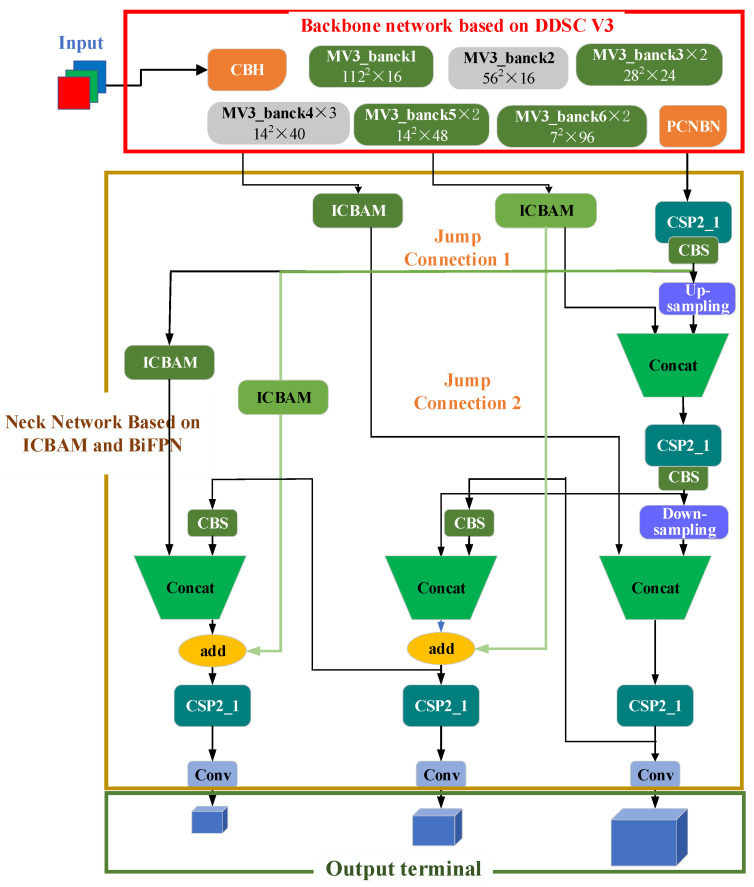
Model diagram of the DV3_IBi_YOLOv5s algorithm.

Combining the improved backbone network and neck network, a vehicle detection algorithm based on a lightweight backbone network and multiscale neck network, namely, DV3_Ibi_YOLOv5s, is proposed to maximize the vehicle detection performance and meet the actual requirements of higher requirements for vehicle detection tasks, as shown in Figure 4.

### 4.1. Backbone Network Based on DDSC_V3

In Figure 4, the red line frame shows the backbone network based on DDSC_V3, which replaces the backbone network of YOLOv5s with the MobileNetv3 network and improves the deep separable convolution in MobileNetv3 to deep hole separable convolution to improve the accuracy of feature detection.

According to the needs of YOLOv5s, the output structure of Mobilenetv3 is improved. The improved MobileNetv3 exists in the form of a feature extractor and outputs multiple feature maps. The MV3_bank2, MV3_bank4, and pcnbn modules are used as outputs to match the neck network of the YOLOv5 satellites.

### 4.2. Neck Network Based on ICBAM and BiFPN

In Figure 4, the blue wireframe is the neck network based on the IBI. The network takes the three-dimensional characteristic diagrams output by the backbone network based on DDSC_V3 as the input, and the icbam attention mechanism module is added after the output characteristic diagrams of the MV3_bank2 and MV3_bank4 modules to obtain the important characteristics of vehicle information. In the figure, the FPN in YOLOv5S is replaced by the high-speed bifpn module, and the icbam attention mechanism module is added in the process of the bifpn jump connection to improve the network’s ability to represent different scales and semantic features to improve the detection accuracy of vehicle targets of various sizes and categories.

This article combines an improved backbone network and a neck network and proposes a vehicle detection algorithm based on a lightweight backbone network and a multiscale neck network, namely, DV3_IBi_YOLOv5s, which aims to maximize the vehicle detection performance and meet the actual requirements of higher requirements for vehicle detection tasks. Figure 4 shows a schematic diagram of the DV3_IBi_YOLOv5s model proposed in this article, which is divided into three main parts: the backbone network based on DDSC_V3, the neck network based on icbam and bifpn, and the output terminal.

## 5. Experimental Results and Analysis

### 5.1. Experimental Data

In the experiment, this article selects the Ua-Detrac dataset to verify the effectiveness of the algorithm. The dataset contains information on cars, trucks, and buses, with a total of 140,000 images and 8250 manually labeled vehicles. The data-computing environment used in this paper is the Windows 10 operating system, the CUDA version is 11.7, and the graphics card model is a GeForce RTX 3080. Python version 3.7 is selected as the network development framework, and the whole development process is completed in the PyCharm integrated development environment. In this dataset, vehicles are divided into four categories, namely, cars, buses, vans, and others. Weather is divided into four categories: cloudy, night, sunny, and rainy. The dataset scale is based on the square root of the area (in pixels), which is divided into three levels: small (0–50 pixels), medium (50–150 pixels), and large (more than 150 pixels). The degree of occlusion is defined according to the score of the occluded vehicle bounding box, which is divided into three categories: no occlusion, partial occlusion (1–50%), and severe occlusion (more than 50%). The cutoff rate is defined as the extent to which the vehicle components exceed the frame.

**Figure 5:**
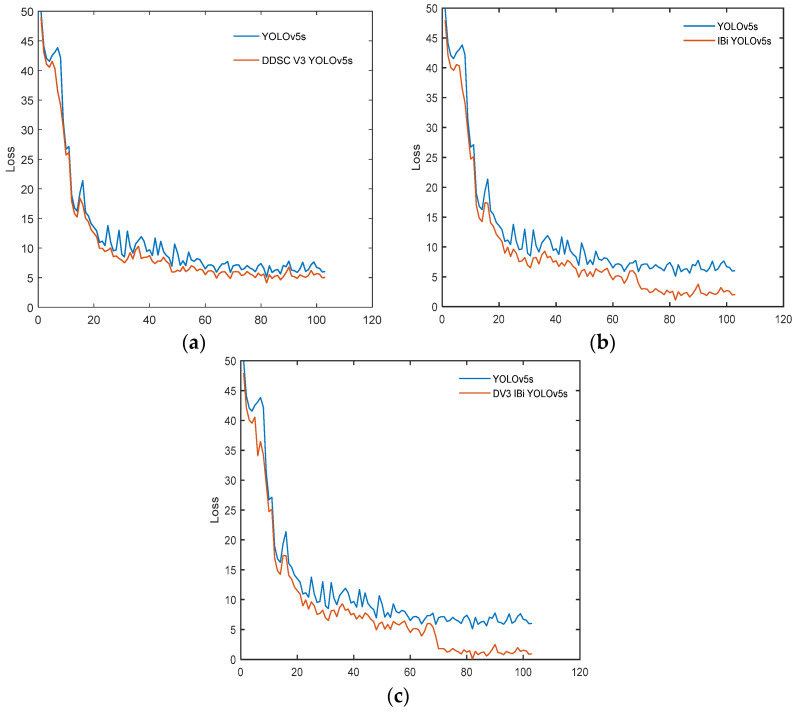
Change curve of the algorithm loss function. (**a**) Loss curve of DDSC_V3_YOLOv5s. (**b**) Loss curve of IBi_YOLOv5s. (**c**) Loss curve of DV3_IBi_YOLOv5s.

To maintain consistency, the number of iterations is 100, and the batch size is 16. The DDSC_V3_YOLOv5s algorithm iteratively trains for 100 epochs in total, and the change curves of the training loss function and the verification loss function are shown in Figure 5. The IBi_YOLOv5s algorithm iteratively trains for 100 epochs. The loss function loss curve of IBi_ YOLOv5s is shown in Figure 5. DV3_IBi_YOLOv5s was iteratively trained 100 times, and the change curves of its training loss function and verification loss function are shown in Figure 5.

As shown in Figure 5a, the loss function curve of the YOLOv5 algorithm after adding lightweight DDSC_V3 is smoother. In the first 10 epochs, the loss function decreased rapidly and then tended to stabilize without obvious fluctuations, indicating that the improved model is more stable and has better convergence. As shown in Figure 5b, during the first 10 epochs of training, the loss function curve showed an exponential downward trend, while the later region tended to stabilize, but when 70 epochs were used, the loss function curve declined rapidly again and finally tended to converge and no longer fluctuated significantly. As shown in Figure 5c, the observation curve shows that the loss function decreases rapidly within the initial 5 epochs and tends to converge and remain relatively stable after 70 epochs.

### 5.2. Ablation Experiment

To verify the effectiveness of the proposed algorithm, ablation experiments are carried out on each module.

(1) Ablation Experiment of the Mobilenetv3 module

The effectiveness of the MobileNetv3 module based on DDSC (DDSC_V3 for short), a component of DDSC_V3_YOLOv5s, was verified by ablation experiments.

As shown in Table 1, the introduction of the MobileNetv3 module improves the detection speed by 3.59% and reduces the detection accuracy by 1.75% compared with those of the benchmark algorithm yolov5s on the Ua-detrac dataset. Compared with that of the MobileNetv3 model, the detection accuracy of the DDSC_V3 module is improved by 5.20%, and the detection speed is improved by 11.69%. Compared with that of the benchmark algorithm YOLOv5s, the detection accuracy is reduced by 1.75% and the detection speed is increased by 15.32%. The integration of Mobilenetv3 and ddsc can improve the detection speed without increasing the detection accuracy. Experiments have proven the effectiveness of the DDSC_V3 module proposed in this article.

(2) Ablation Experiment of the BiFPN_ICBAM module

The ICBAM attention mechanism, a component of IBi_YOLOv5s, and the effectiveness of the BiFPN_ICBAM module were verified by ablation experiments.

As shown in Table 2, compared with the benchmark algorithm YOLOv5s, when the FPN is improved by adding an attention mechanism, the detection accuracy increases and the tracking speed decreases. The best combination algorithm is the combination of the bifpn714;icbam algorithms. Compared with that of the benchmark algorithm YOLOv5s, the tracking accuracy is improved by 5.29%, and the detection speed is 96.19 fps. Experiments show that the optimization scheme of icbam and icbam BIFPN proposed in this article is effective.

(3) Ablation Experiment of DDSC_V3 and the IBi module

To verify the effectiveness of the components of the algorithm DV3_IBi_YOLOv5s in this article, ablation experiments were carried out on the DDSC_V3, and the IBi module was verified via ablation experiments.

As shown in Table 3, compared with the benchmark algorithm yolov5s, the introduction of the ddsc_v3 module in the ua-detrac dataset increased the detection speed by 3.59% and reduced the detection accuracy by 1.75%. Compared with the benchmark algorithm, the introduction of the IBi module reduced the detection speed by 11.19% and improved the detection accuracy by 4.29%. Compared with the benchmark algorithm, the simultaneous introduction of the DDSC_V3 module and the IBI module improved the detection accuracy by 7.34% and the detection speed by 10.80%. Ablation experiments demonstrate the effectiveness of the proposed optimization scheme for backbone and neck networks.

### 5.3. Analysis of Algorithm Effectiveness

In this section, in the Car, Truck, and Bus categories of the UA-DETRAC dataset, the detection performance of YOLOv5s is compared with that of the DV3_IBi_YOLOv5s algorithm, DDSC_V3_YOLOv5s algorithm, and IBi_YOLOv5s algorithm proposed in this article.

(1) Performance evaluation results

To verify the performance of the algorithm, the accuracy (Precision (%)), recall (%), accuracy (AP (%)), average accuracy (mAP (%)), F1 score, parameter count (MB), and FPS (frame/s) of DV3-IBi_YOLOv5s will be compared with other algorithms in the Car, Truck, and Bus categories. Firstly, in order to analyze the algorithm performance, DV3-IBi_YOLOv5s was compared with several improved algorithms such as YOLOv5s, DDSCV3_YOLOv5s, IBi_YOLOv5s, etc., in Precision, Recall, and AP. At the same time, we will compare the algorithm with several traditional methods such as YOLOv6, YOLOv7, YOLOv8, SSD, and Faster RCNN to verify the performance of this method. The experimental results are shown in Table 4.

As shown in Table 4, the average values of the precision, recall, and AP of the YOLOv5s, DDSC_V3_YOLOv5s, IBi_YOLOv5s, and DV3_IBi_YOLOv5s algorithms for the three vehicle detection classifications are 82.77%, 53.43%, and 66.49%; (62%, 52%), 65%, and 65.17%; 86%, 54.23%, and 69.81%; and 86.02%, 54.39%, and 71.19%, respectively. Compared with the benchmark algorithm YOLOv5s, the DV3_IBi_YOLOv5s proposed in this article improved the overall precision, recall, and AP values by 3.93%, 1.79%, and 7.34%, respectively. Compared with those of the other two single improved models, the overall precision, recall, and AP of the DV3_IBi_YOLOv5s model proposed in this article are significantly better.

Secondly, to verify the performance of this algorithm in F1 score evaluation metrics, DV3-IBi_YOLOv5s was compared with other algorithms in F1 score evaluation metrics, as shown in Table 5.

To verify the performance of this algorithm in mAP, parameter quantity, and FPS evaluation metrics, DV3-IBi_YOLOv5s was compared with other algorithms in F1 score evaluation metrics, as shown in Table 6.

As shown in Table 6, the DV3_IBi_YOLOv5s algorithm in this article increased the map value by 7.34% and reduced the number of parameters by 47.95% compared with those of the YOLOv5s algorithm; moreover, the detection speed increased by 10.80%. The data in the table show that the DV3_IBi_YOLOv5 algorithm in this article achieves a high map value. Although it is inferior to DDSC_V3_YOLOv5s in terms of parameter quantity and detection speed, it is basically flat. The experimental results fully verify the effectiveness of the algorithm in improving the accuracy and speed of vehicle detection.

(2) Lightweight network visualization results

**Figure 6:**
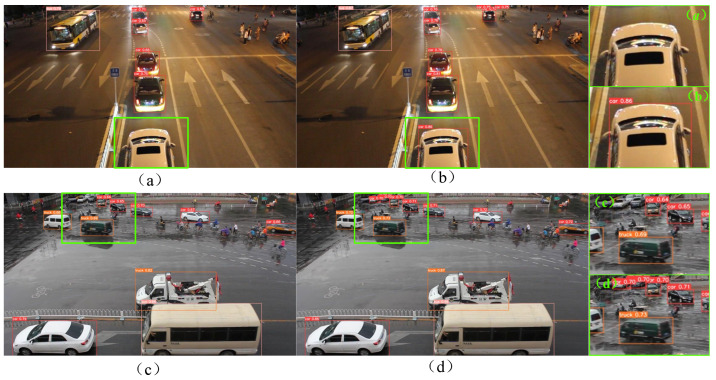
Comparison of experimental results: YOLOv5s (**left**); DDSC_V3_YOLOv5s (**right**). (**a**–**d**) represent vehicle detection scenarios in different situations.

Figure 6 shows a representative visual image of the detection results. The left side of Figure 6 shows the test results of YOLOv5s, and the right side shows the test results of DDSC_V3_ YOLOv5s. Among them, (a)–(d) represent vehicle detection scenarios in different situations.Figure 6a,b shows vehicle detection in a dark environment. Compared with DDSC_V3_YOLOv5s, YOLOv5s in the figure has missed detections and failed to correctly identify the vehicle at the bottom of the image. In this scenario, DDSC_V3_YOLOv5s correctly identified the car, and its confidence reached 0.86, indicating that the detection accuracy of the improved DDSC_V3_YOLOv5s will not be affected in the case of insufficient light. By comparing the detection results of Figure 6c,d, it can be found that the problem of missing detection of small target vehicles at a distance in Figure 6c is significant, while the detection effect of Figure 6d is relatively good, and it can capture distant vehicle targets, although the confidence is still not high enough.

(3) Multiscale network visualization results

**Figure 7:**
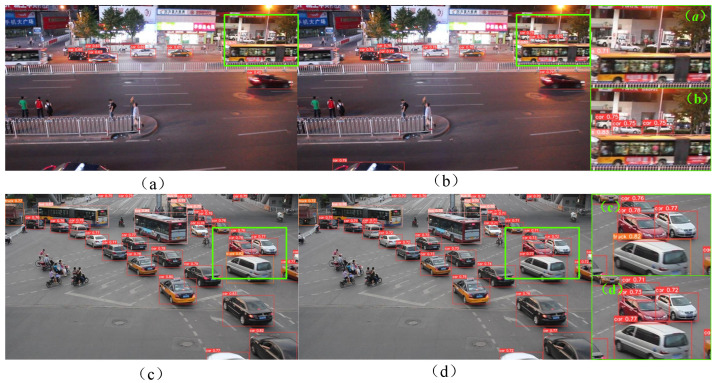
Comparison of detection results between YOLOv5s and IBi_YOLOv5s algorithms in dim lighting environments at night and street environments with a large number of vehicles: YOLOv5s (**left**); IBi_YOLOv5s (**right**). (**a**–**d**) represent vehicle detection scenarios in different situations.

Figure 7 shows a comparison of the detection results of the YOLOv5s and IBi_YOLOv5s algorithms in a dark lighting environment at night and a street environment with a large number of vehicles. Among them, (a)–(d) represent vehicle detection scenarios in different situations. In Figure 7a,b, IBi_YOLOv5s can more accurately detect the bus on the left and small car-like targets at a distance in a complex environment than YOLOv5s can. Visualization results show that, to a certain extent, the IBi_YOLOv5s algorithm is better than the original YOLOv5s algorithm for vehicle detection in complex environments. In Figure 7c,d, IBi_YOLOv5s successfully located and correctly classified all vehicles, while YOLOv5s successfully located all vehicles but did not correctly classify them. Specifically, in Figure 7c, Truck is incorrectly classified as the Car category, which verifies that IBi_YOLOv5s has more advantages than the original YOLOv5s algorithm in terms of classification of detection targets.

**Figure 8:**
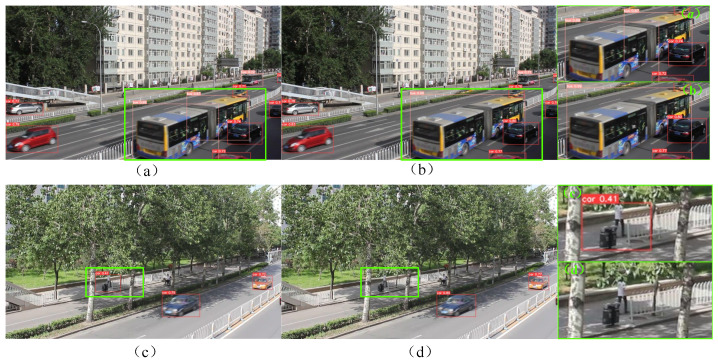
Comparison of detection results between YOLOv5s and IBi_YOLOv5s algorithms in normal environments: YOLOv5s (**left**); IBi_YOLOv5s (**right**). (**a**–**d**) represent vehicle detection scenarios in different situations.

In Figure 8, the left side shows the test results of YOLOv5s and the right side shows the test results of the IBI_YOLOv5s proposed in this article. Compared with Figure 8a,b, it can be observed that the bus is not correctly detected and located in (a), but one vehicle is mistakenly detected as two buses. In contrast, Figure 8b has high confidence and can more accurately detect and locate the bus information in the image. The results show that the performance of IBi_YOLOv5s in the feature perception of bus vehicles is better than that of the original YOLOv5s algorithm to a certain extent. Compared with Figure 8c,d, the recognition rate of the car in Figure 8c is not high. Garbage cans in roadside shadows are incorrectly recognized as vehicles, and the recognition confidence of cars is slightly lower than that in Figure 8d. The results show that the vehicle detection performance of IBi_YOLOv5s is better than that of the original YOLOv5s algorithm, especially in the shadowed area.

(4) Lightweight multiscale network visualization results

**Figure 9:**
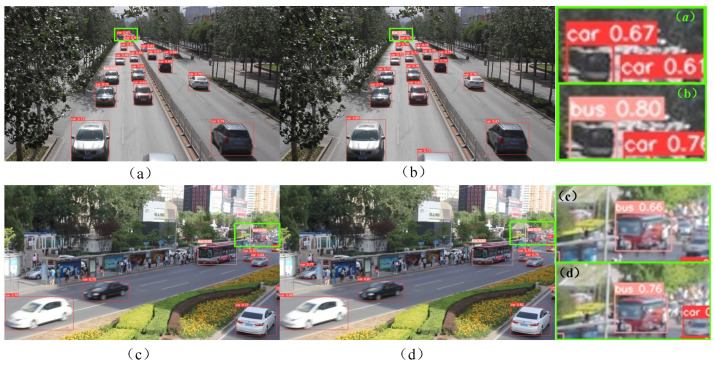
Comparison of the experimental results: YOLOv5s (**left**); DV3_IBi_YOLOv5s (**right**). (**a**–**d**) represent vehicle detection scenarios in different situations.

In Figure 9, the left side shows the detection results of YOLOv5s, while the right side shows the detection results of DV3_IBi_YOLOv5s. Among them, (a)–(d) represent vehicle detection scenarios in different situations. Comparing Figure 9a,b, it can be found that both can identify small-scale and numerous car targets on the street. However, there is false detection in Figure 9a; for example, the bus category on the left is falsely detected as a car category. To some extent, the DV3_IBi_YOLOv5s model has better feature extraction ability and a better detection effect. On the other hand, the comparison between Figure 9c,d shows that the detection frame in Figure 9c only recognizes part of the structure and does not completely frame the bus, while the detection frame size in Figure 9d is more accurate and closer to the actual size of the bus. Compared with the above results, the detection effect of DV3_IBi_YOLOv5s is better, and the target positioning is more accurate in the target detection task.

The experimental data strongly verify that the multistage improvement of the YOLOv5s algorithm in this paper has a substantial effect. Compared with the original YOLOv5s algorithm, the performance improvement of DV3_IBi_YOLOv5s is not only reflected by quantitative data but can also be displayed by visual images through a comparison of the prediction results. This means that the application of the DV3_IBi_YOLOv5s algorithm in actual scenes will significantly improve the speed and accuracy of vehicle detection, thus greatly improving the overall effect of vehicle detection.

## 6. Conclusions

To balance the detection accuracy and speed of YOLOv5s in real-time vehicle detection tasks, this article proposes a vehicle detection algorithm based on a lightweight backbone network and a multiscale neck network. First, Mobilenetv3 is introduced into the backbone network to replace the original backbone network. On this basis, a deep space separable convolution is built. Then, an ICBAM mechanism is built between the backbone network and the neck network. Finally, an efficient two-way cross-scale connection BiFPN is added to the neck network, and the ICBAM attention mechanism is integrated into the hopping connection of bifpn. The effectiveness of the algorithm in this article is verified on the UA-DETRAC dataset. Compared with YOLOv5s, the accuracy, recall, and map value of DV3_IBi_YOLOv5s in this article are improved by 3.93%, 1.79%, and 7.34%, respectively. In terms of parameter quantity, it is reduced by 47.95%, and the detection speed is increased by 10.80%. The algorithm in this article effectively improves the detection accuracy and speed of YOLOv5s.

## Figures and Tables

**Table 1:** Experimental results of DDSC_V3_YOLOv5s ablation.

YOLOv5s	MobileNetV3	DDSC_V3	mAP/%	FPS/Frame/s
✓			66.32	108.32
	✓		62.13	112.36
		✓	65.16	124.91

**Table 2:** Experimental results of BiFPN_ICBAM ablation.

YOLOv5s	CBAM	ICBAM	BiFPN	BiFPN_ICBAM	mAP (%)	FPS (Frame/s)
✓					66.3	108.32
	✓				67.22	99.23
		✓			68.77	103.21
			✓		68.69	106.19
				✓	69.32	101.02
	✓		✓		69.42	98.71
	✓			✓	69.72	96.00
		✓	✓		69.69	98.32
		✓		✓	69.81	96.19

**Table 3:** Experimental results of DV3_IBi_YOLOv5s ablation.

YOLOv5s	DDSC_V3	IBi	mAP/%	FPS/frame/s
✓			66.32	108.32
	✓		65.16	124.91
		✓	69.81	96.19
	✓	✓	71.19	120.02

**Table 4:** Comparison between DV3_IBi_YOLOv5s and other algorithms in terms of precision, recall, and AP.

Model	Precision (%)			Recall (%)			AP (%)		
	Car	Bus	Truck	Car	Bus	Truck	Car	Bus	Truck
YOLOv5s	83.98	90.38	73.97	50.23	71.03	39.03	67.52	81.38	50.56
DDSC_V3_YOLOv5s	83.56	89.02	72.59	49.99	69.54	38.42	66.61	79.86	49.03
IBi_YOLOv5s	86.39	93.81	77.80	52.13	71.14	39.42	71.30	84.10	54.03
DV3_IBi_YOLOv5s	86.41	92.90	78.75	52.17	71.24	39.76	73.21	83.93	56.44
YOLOv6	80.23	88.00	70.26	46.20	55.36	33.62	60.32	79.22	44.20
YOLOv7	82.11	89.12	72.19	47.22	59.55	37.55	65.67	77.32	45.99
YOLOv8	82.36	90.66	74.28	46.28	59.68	35.67	66.33	82.45	49.69
SSD	79.83	77.28	70.21	33.56	50.21	30.22	57.54	72.13	48.22
Faster-RCNN	80.66	78.33	72.33	39.21	52.02	30.85	59.58	76.89	49.55

**Table 5:** Comparison of F1 score evaluation metrics between DV3-IBi_YOLOv5s and other algorithms.

Model	F1		
	Car	Bus	Truck
YOLOv5s	0.628614	0.795452	0.510982
DDSC_V3_YOLOv5s	0.625558	0.780834	0.502461
IBi_YOLOv5s	0.650233	0.809172	0.523268
DV3_IBi_YOLOv5s	0.6506	0.806409	0.528411
YOLOv6	0.586352	0.679643	0.454783
YOLOv7	0.599588	0.713943	0.494029
YOLOv8	0.592603	0.71978	0.481959
SSD	0.472545	0.608711	0.422532
Faster-RCNN	0.527685	0.625198	0.432522

**Table 6:** Comparison between DV3_IBi_YOLOv5s and other algorithms in terms of mAP, parameter quantity, and FPS.

Model	mAP (%)	Parameter Quantity (MB)	FPS (Frame/s)
YOLOv5s	66.32	7.3	108.32
DDSC_V3_YOLOv5s	65.16	3.5	124.91
IBi_YOLOv5s	69.81	7.5	96.19
DV3_IBi_YOLOv5s	71.19	3.8	120.02
YOLOv6s	61.25	17.19	118.23
YOLOv7-tiny	62.99	6.01	128
YOLOv8s	66.16	11.2	125.52
SSD-300	59.30	140	59
Faster-RCNN-16	62.01	148	12

## Data Availability

Data are contained within the article.
